# Polyaminoacid Based Core@shell Nanocarriers of 5-Fluorouracil: Synthesis, Properties and Theranostics Application

**DOI:** 10.3390/ijms222312762

**Published:** 2021-11-25

**Authors:** Marta Szczęch, Alicja Hinz, Natalia Łopuszyńska, Monika Bzowska, Władysław P. Węglarz, Krzysztof Szczepanowicz

**Affiliations:** 1 Jerzy Haber Institute of Catalysis and Surface Chemistry, Polish Academy of Sciences, 30-239 Krakow, Poland; marta.anna.szczech@gmail.com; 2 Faculty of Biochemistry, Biophysics and Biotechnology, Jagiellonian University, 30-387 Krakow, Poland; alicja.hinz@uj.edu.pl (A.H.); monika.bzowska@uj.edu.pl (M.B.); 3 Henryk Niewodniczański Institute of Nuclear Physics, Polish Academy of Sciences, 31-342 Krakow, Poland; natalia.lopuszynska@ifj.edu.pl (N.Ł.); Wladyslaw.Weglarz@ifj.edu.pl (W.P.W.)

**Keywords:** nanocarriers, 5-Fluorouracil, theranostics, drug delivery, anticancer, fluorine magnetic resonance imaging

## Abstract

Cancer is one of the most important health problems of our population, and one of the common anticancer treatments is chemotherapy. The disadvantages of chemotherapy are related to the drug’s toxic effects, which act on cancer cells and the healthy part of the body. The solution of the problem is drug encapsulation and drug targeting. The present study aimed to develop a novel method of preparing multifunctional 5-Fluorouracil (5-FU) nanocarriers and their in vitro characterization. 5-FU polyaminoacid-based core@shell nanocarriers were formed by encapsulation drug-loaded nanocores with polyaminoacids multilayer shell via layer-by-layer method. The size of prepared nanocarriers ranged between 80–200 nm. Biocompatibility of our nanocarriers as well as activity of the encapsulated drug were confirmed by MTT tests. Moreover, the ability to the real-time observation of developed nanocarriers and drug accumulation inside the target was confirmed by fluorine magnetic resonance imaging (^19^F-MRI).

## 1. Introduction

Cancer is a leading cause of death worldwide, accounting for nearly 10 million deaths in 2020 (World Health Organization). Despite significant progress in cancer research, it remains a disease with limited treatment approaches. Metastasis and recurrence of cancer contribute a lot to disability and mortality. Currently, the issue is superior due to the soaring mortality rates correlated with the COVID-19 pandemic situation and a rapidly aging population. Global Cancer Observatory predicts that by 2030 approximately 30 million cancer patients will die from cancer each year. Standard cancer treatment is invasive and consists of surgical resection, chemotherapy, and radiotherapy. Surgery is an effective measure to remove solid malignant tumors, especially in an early stage of cancer development; however, due to late diagnosis, combined therapy involves surgery, chemotherapy, and radiotherapy is necessary for treatment. The disadvantages of chemotherapy are the toxic side effects and the development of resistance to the chemical agents, which is related to non-specific targeting of chemotherapeutics that harming both healthy cells and cancer ones, moreover, poor drugs bioavailability in biological fluids, undesirable side effects, and faint drugs loading capacity resulting in low therapeutic effectiveness [1]. Although novel approaches are constantly proposed, there are still many obstacles to overcome. The main challenge is to find new, more sophisticated, and efficient drug delivery systems capable of overcoming all of the limitations through the delivery of chemotherapeutics only to strictly defined pathologically changed places in the body. Details concerning targeted drug delivery systems as well as targeting moieties can be found in related reviews [2,3,4,5,6,7]. Among drug delivery systems, polymeric nanocarriers for passive targeting based on the EPR effect are intensively investigated [5]. The next step in developing nanocarriers is to engineer nanocarriers, which can demonstrate a combination of various functions, i.e., to construct multifunctional drug nanocarriers [8,9]. Despite the therapeutic role of nanocarriers, moiety allowing for the real-time observation of its accumulation inside the target is required. The combination of therapeutic and diagnostic functions in one system is called theranostics [10]. Various non-invasive in vivo imaging techniques are available for diagnoses, such as magnetic resonance imaging (MRI), positron emission tomography (PET), computed tomography (CT), single-photon emission computed tomography (SPECT), ultrasound (US), and optical imaging (OI). Magnetic resonance imaging has become a powerful tool for non-invasive imaging due to its high spatial resolution [11].

Magnetic resonance imaging produces tomographic images, but contrary to CT, it does not utilize ionizing radiation. MR images are generated by the excitation of nuclei using RF pulses followed by a spatial encoding of the nuclear magnetic resonance (NMR) signal using gradient fields [12]. Routine MR imaging is performed on the ^1^H nucleus due to its high abundance in the body. However, other nuclei with nonzero spin, e.g., ^19^F, can also be used in MR experiments [13,14]. For investigations concerning drugs biodistribution and accumulation, magnetic resonance imaging and spectroscopy on ^19^F nuclei have brought much interest [15,16,17]. There are several properties of ^19^F nucleus that make it an excellent tracer for in vivo imaging of exogenous substances. Fluorine-19 has I = ½ spin, and its gyromagnetic ratio γ has a value similar to ^1^H (40.06 MHz/T for ^19^F vs. 42.58 MHz/T for ^1^H), which means, that it resonates at the frequency close to the one of ^1^H (ω = γ∙B_0). Therefore, standard ^1^H instruments can be used with only minor modifications, which is essential for clinical applications. Moreover, ^19^F isotope has 100% natural abundance, and its relative sensitivity is equal to 0.83. Another feature that is important for drug distribution observation, is that ^19^F almost does not appear physiologically in the human body, which provides an excellent contrast-to-noise ratio and specificity for the exogenous ^19^F compounds [18].

5-fluorouracil (5-FU) is one of the few rationally designed chemotherapeutics used to treat various cancers [19,20,21]. 5-FU is an analog of uracil with a fluorine atom at the C-5 position in place of hydrogen [19,20]. The mechanism of action of 5-FU is related to inhibiting nucleoside metabolism and DNA synthesis [20,22,23,24]. Nevertheless, clinical use of 5-FU is limited due to a short half-life, systemic toxicity, and non-selective delivery [23,25]. Therefore, it is essential to develop effective multifunctional 5-FU nanocarriers to improve targeted delivery, resulting in improved anticancer treatments. The following nanocarriers of 5-FU were proposed: polymer-based carriers such as chitosan beads, Eudragit^®^, Poly(alkylcyanoacrylates), PLGA, β-cyclodextrin as well as liposomes, nanoemulsions, solid lipid nanoparticles, gold nanoparticles, silica nanocarriers, iron oxides nanoparticles, graphene oxides, and QD [24,25,26,27,28,29,30,31,32,33,34,35]

The present contribution aimed to develop a novel method of preparing multifunctional 5-FU nanocarriers and their in vitro characterization. 5-FU polyaminoacid-based core@shell nanocarriers were formed by the encapsulation of drug-loaded nanocores with polyaminoacids multilayer shell via the layer-by-layer method with saturation technique. Biocompatibility of nanocarriers and activity of encapsulated 5-FU were evaluated. Moreover, fluorine magnetic resonance imaging (^19^F-MRI) confirmed the ability to the real-time observation of developed nanocarriers and drug accumulation inside the target.

## 2. Results and Discussion

### 2.1. Optimization, Characterization, and Stability of 5-Fluorouracil Nanocarriers

Encapsulation of liquid cores with a polyelectrolyte multilayer shell was initially developed for encapsulation of hydrophobic, water-insoluble actives (or low solubility in water). In contrast, here, we expand this method to the encapsulation of sparingly soluble active substances. 5-Fluorouracil (5-FU) is sparingly soluble in water, slightly soluble in alcohol, and practically insoluble in chloroform (data from National Center for Biotechnology Information. PubChem Compound Summary for CID 3385, 5-Fluorouracil. https://pubchem.ncbi.nlm.nih.gov/compound/5-Fluorouracil, accessed on 18 October 2021). 5-FU is an anticancer drug used to treat of multiple solid tumors; however, its short biological half-life and little affinity to tumor cells limit therapeutic efficacy. Undoubtedly, an additional benefit of 5-FU is the fluorine atom contained in the compound structure, which allows the possibility to monitor that drug by MRI [36,37,38]. Taking into account all this information, 5-FU was selected for our investigation.

**Figure 1:**
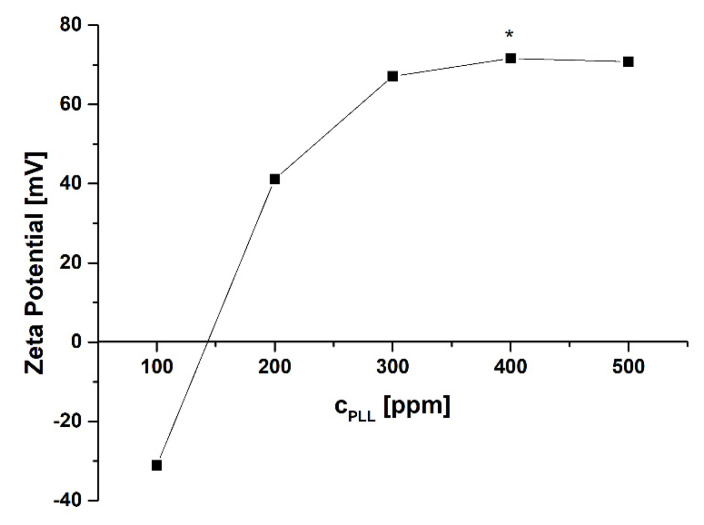
Changes of the zeta potential of prepared nanocores with PLL concentration (PLL—poly(L-lysine), * optimal concentration of PLL).

The suspension of nanocores containing 5-FU was prepared by adding 0.1 mL of 5-FU and anionic surfactant AOT dissolved in DMSO to the 50 mL of polycation solution (cationic polyaminoacid PLL) during stirring by a magnetic stirrer. The anionic surfactant AOT with polycation can form a stable interfacial complex [39] used to form and stabilize nanocore suspension. For that preparation, the AOT to PLL ratio has to be optimized to minimize the free unabsorbed polyelectrolyte (PLL) in nanocore suspension, which is crucial for further encapsulation via the layer-by-layer method. AOT concentration (330 mg/mL) was chosen according to our previous studies [39,40], whereas PLL concentration was determined experimentally. The following concentrations of PLL were tested 100, 200, 300, 400, 500 ppm, and the optimal one was selected by analyzing zeta potential and stability of formed nanocores. It corresponds to the point after overcharging and directly before reaching the plateau of the value of zeta potential dependence versus concentration of cationic polyaminoacid PLL (Figure 1).

**Figure 2:**
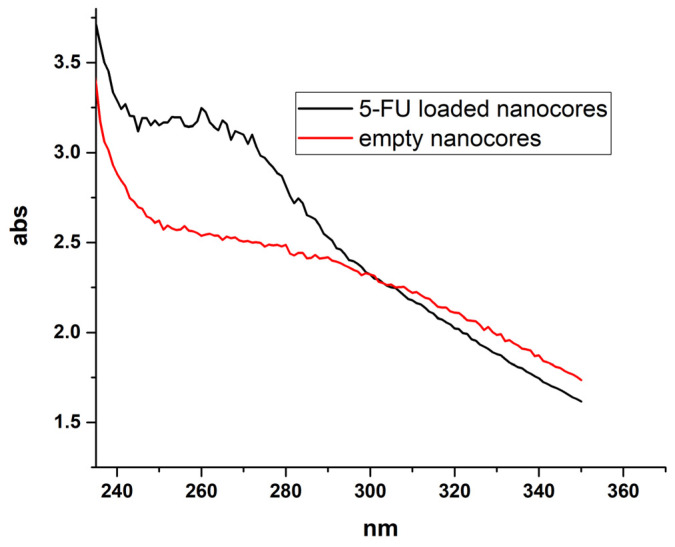
The comparison of UV–Vis spectra of empty and 5-FU loaded nanocarriers provided as evidence of successful encapsulation.

The average size nanocores prepared with an optimal concentration of PLL was 80 nm with a polydispersity index (PDI) of below 0.2. At the same time, the relatively high value of zeta potential +72 ± 10 mV ensures electrostatic stabilization of the colloidal nanosystem. After forming 5-FU loaded nanocores, the suspension was separated by ultrafiltration (Amicon Ultra MWCO 3000) and drug content in the supernatant was measured. Calculated encapsulation efficiency was 53%, while a comparison of spectra of empty and 5-FU loaded nanocarriers provided evidence of successful encapsulation of the drug (Figure 2).

**Figure 3:**
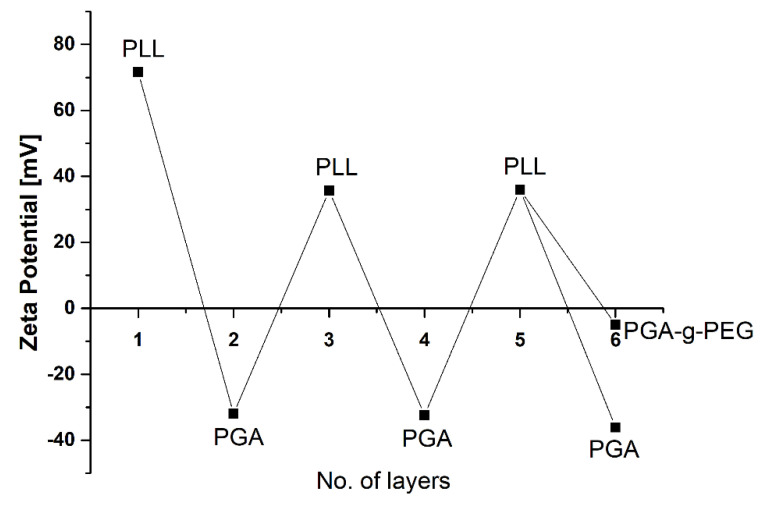
Dependence of the zeta potential of polyaminoacid core-shell nanocarriers on the adsorption of subsequent polyelectrolytes’ layers (PLL—Poly(L-lysine), PGA—poly(l-glutamic acid), and PGA-g-PEG Poly(l-glutamic acid)-g-poly(ethylene glycol).

The positively charged 5-FU loaded and empty nanocores were further encapsulated using the layer-by-layer method (LbL) with the saturation technique. For LbL encapsulation, two oppositely charged polyaminoacids PLL (2 mg/mL in 0.015 M NaCl) and PGA (2 mg/mL in 0.015 M NaCl) were selected. In contrast, PGA-g-PEG (200 mg/mL) was used to form an external layer in a multilayer shell. Such pegylated polyaminoacid allows the formation of pegylated nanocarriers. The main evidence on the formation of the multilayer structure is shown Figure 3, where the typical dependence of nanocarrier zeta potential on adsorption of consecutive polyaminoacid layers is presented.

Characterization of our 5-FU nanocarriers is summarized in Table 1. The size of developed 5-FU nanocarriers fulfills the literature requirements to appear to be appropriated for drug delivery systems by the occurrence of enhanced permeability and retention effect (EPR effect). Moreover, functionalization with PGA and PEG restricts or even eliminates the non-specific binding of proteins and immune cells after iv administration, which should ensure circulating for a long time, and which is necessary for a sufficient level of accumulation in the tumor [41,42,43,44].

### 2.2. Magnetic Resonance Imaging

**Figure 4:**
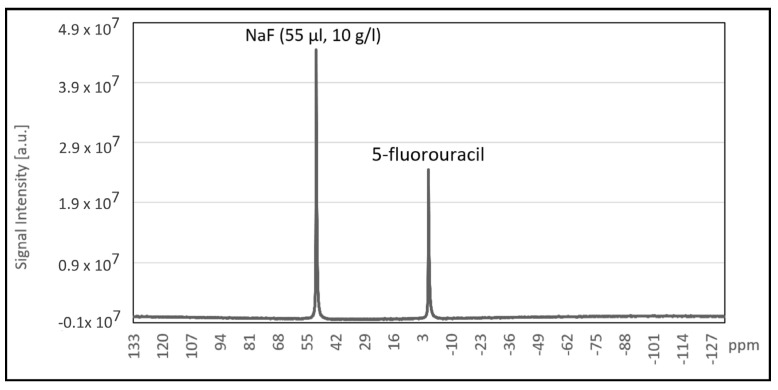
^19^F spectroscopy of the suspension of 5-FU loaded nanocarriers.

Quantitative ^19^F NMR spectroscopy for the suspension of 5-FU NC 1 nanocarriers was performed with added 55 µL of 10 g/L solution of NaF in distilled water as a standard (Figure 4). Based on differences in peak integrals, the content of ^19^F nuclei in 5-FU loaded nanocarriers and the concentration of 5-FU in the sample were calculated. Peak observed for 5-FU corresponds to 4.55 × 10^18 19^F nuclei which is equivalent of 982.73 mg/L 5-FU concentration. For T_1_ calculation, points measured in the inversion recovery experiment were fitted with the function: $\left(t\right)=I_{0}\left(1-2e^{\left(-\frac{t}{T1}\right)}\right)$, resulting in T_1_ = 3.23 ± 0.12 s.

**Figure 5:**
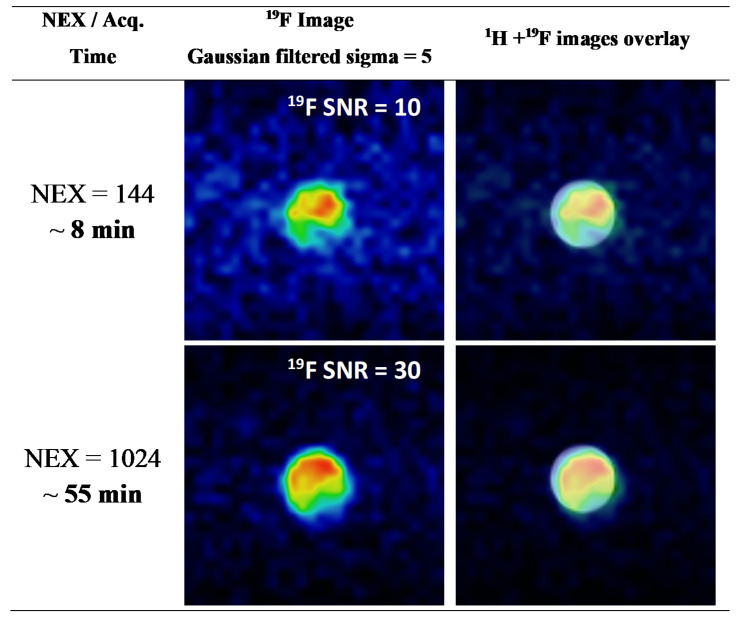
^19^F images of the sample containing nanocarriers loaded with 5-fluorouracil (5-FU NC 1 PGA).

In Figure 5, two cases are presented. The first one was obtained with a reasonable SNR value equal to ~10, which is already enough to reliably determine 5-FU nanocarriers’ location, with a total acquisition time of 7 min and 40 s. In the second one the increasing number of acquisitions and total acquisition time to 54 min and 36s, resulted in SNR up to 30. This result can be treated as a proof of concept of utilizing ^19^F MRI for 5-FU detection and it constitutes the first step of investigation for biomedical application which is mandatory for the planning of any in vitro and in vivo studies. Shepelytskyi [45], Otake, K. Hirata [46], and Doi [47] et al. reported successful in vivo detection of localized ^19^F signal, not only of 5-FU but also for its metabolites after the injection of 5 FU at a dose of 455–550 mg/kg (at 3.0 T field), 250 mg/kg (at 7.0 T field) and 130–260 mg/kg (9.4 T field) respectively. As the SNR scales linearly with ^19^F dose, we expect that imaging with the dose in a range that is usually used in animal studies on small rodents (100–300 mg/kg), would still produce sufficient SNR (SNR = 3–10) to evaluate the 5-FU nanocarriers distribution within 30 min timeframe.

### 2.3. Bioanalysis

**Figure 6:**
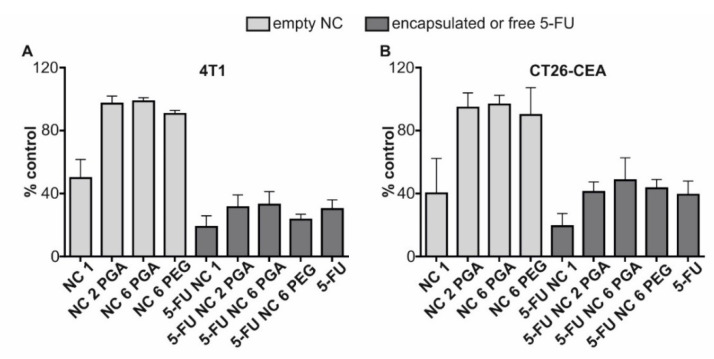
The effect of free and encapsulated 5-fluorouracil on the viability of murine cancer cells 4T1 (**A**) and CT26-CEA (**B**). The cells were incubated 48 h with empty nanocarriers, free or encapsulated 5-fluorouracil. MTT analysis was performed to assess the cell viability. The error bars represent the mean ± SD of at least three independent experiments using nanocarriers derived from three different syntheses.

Encapsulated 5-fluorouracil retains toxic activity against murine cancer cells. The biological activity of encapsulated fluorouracil was verified on murine cancer cell lines: 4T1 and CT26-CEA. The cells were exposed for 48 h on empty nanocarriers, encapsulated 5-FU, and free 5-FU, followed by viability analysis. As presented in Figure 6, it was confirmed that encapsulated 5-FU exhibits toxicity comparable to free 5-FU. 4T1 cells were more sensitive to its effect; free and encapsulated fluorouracil caused a decrease in viability to about 30%. In the case of CT26-CEA cells, the viability decreased to about 40%. For 5-FU NC1, it was impossible to determine antitumor activity because even positively charged and empty NC1 nanocarriers were toxic to the cancer cells [48,49].

**Figure 7:**
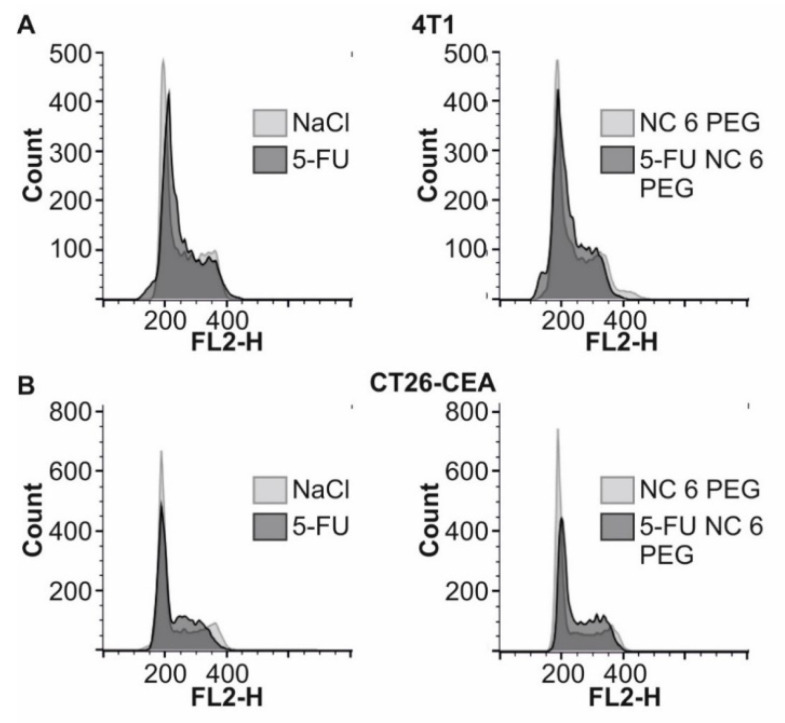
Changes in the cell cycle progression induced by free or encapsulated 5-fluorouracil. 4T1 cells (**A**) and CT26-CEA cells (**B**) were exposed for 48h to free 5-fluorouracil or 5-FU NC 6 PEG. Next, the cells were collected from plates, stained with PI, and the flow cytometry analysis was performed to analyze the changes in the cell cycle. Results represent three independent experiments.

Encapsulated 5-fluorouracil arrests progression of the cell cycle, similarly to free drug Fluorouracil, is an antimetabolite that disrupts DNA replication. Thus, the cell cycle’s progression, causing arrest in the S phase of the cell cycle [50,51]. Analysis of the cell cycle of CT26-CEA and 4T1 cells followed 48h exposition to encapsulated and free fluorouracil was performed using flow cytometry after staining the cellular DNA with propidium iodide. Since toxicity analysis demonstrated similar anticancer activity of 5-FU NC 2 PGA, 5-FU NC 6 PGA, and 5-FU NC 6 PEG in this experiment, we analyzed only the effect of free drug and 5-FU NC 6 PEG. We observed that changes in the cell cycle progression are almost identical for the free drug and 5-FU NC 6 PEG. As shown in Figure 7, the population of 4T1 and CT26-CEA cells representing G1 and G2/M phase of the cell cycle was decreased for 5-FU NC 6 PEG and free 5-FU in comparison to cells incubated with NaCl or NC 6 PEG. At the same time, the population of cells representing the S phase (the amount of DNA in the cell doubles during the S phase and is higher than 2n) is higher for the cells treated with the free or encapsulated drug.

## 3. Materials and Methods

### 3.1. Chemicals

5-Fluorouracil (5-FU) was purchased from Selleck Chemicals, STI, Poznań, Poland. The polyaminoacids: Poly(L-lysine) hydrobromide, PLL (Mw = 15,000–30,000) and poly(l-glutamic acid) sodium salt, PGA (MW 15,000 to 50,000), dimethyl sulfoxide DMSO, and sodium chloride were obtained from Sigma-Aldrich, Poznań, Poland, Surfactant docusate sodium salt (AOT) was purchased from Cytec Solvay, Warszawa, Poland. All materials were used as received without further purification. Poly(l-glutamic acid)-g-poly(ethylene glycol)—a pegylated polyelectrolyte referred as PGA-g-PEG was previously synthesized in our lab [52]. Ultrapure water was produced using a Millipore Direct-Q5 UV purification system.

### 3.2. Nanocarriers Synthesis

5-Fluorouracil core@shell structure nanocarriers were synthesized by adopting the previously developed method [53] with some modification, i.e., encapsulation of liquid cores with polyelectrolyte multilayer shell. The nanocores were prepared by addition of a solution of 5-fluorouracil (45 mg/mL) and AOT (330 mg/mL) in DMSO to the poly-L-lysine solution during stirring by magnetic stirrer (300 RPM). Formed nanocores were encapsulated with polyaminoacids multilayer shell via layer-by-layer method with saturation technique [54,55]. A fixed volume of 5-FU loaded, or empty nanocores, were added to the oppositely charged polyaminoacid’s solution with various concentrations during vigorous mixing, and the consecutive layer formation was followed by the zeta potential measurements. Then, the coating process was repeated with the use of the oppositely charged polyaminoacids. The multilayer shell was constructed from the following polyaminoacids: poly(L-lysine) as a polycation and poly(l-glutamic acid) as polyanion and poly(l-glutamic acid)-g-poly(ethylene glycol) as pegylated polyanion used to form pegylated external layer.

### 3.3. Nanocarrier Characterization

#### 3.3.1. Size, Size Distribution, and Polydispersity Measurements

Size, size distribution, and polydispersity were measured by Dynamic Light Scattering (DLS) technique with a Zetasizer Nano ZS instrument (Malvern-Pananalytical, Malvern, UK). The measurements were performed at 20 °C in 0.015 M NaCl. The DTS Nano software was applied for data evaluation. All measurements were performed in triplicate.

#### 3.3.2. Zeta Potential Measurements

Zeta Potential was measured by Laser Doppler Velocimetry (LDV) method with the Zetasizer Nano ZS instrument (Malvern-Pananalytical, Malvern, UK). The measurements were performed at 20 °C in 0.015 M NaCl. The DTS Nano software was applied for data evaluation. All measurements were performed in triplicate.

#### 3.3.3. Encapsulation Efficiency

To confirm the 5-Fluorouracil encapsulation as well as to evaluate their efficiency UV–Vis spectroscopy was applied. The UV–Vis absorbance measurements were performed using a UV-1800 spectrophotometer (Shimadzu, Kyoto, Japan) while encapsulation efficiency was determined according to the adapted method previously described [56].

#### 3.3.4. Magnetic Resonance Imaging

Both ^19^F MR spectroscopy/relaxation measurements as well as ^1^H and ^19^F imaging were performed at the 9.4 T Bruker Biospec 94/20 research MRI scanner with 210mm bore diameter and high performance actively shielded BGA 12S HP gradient system (675 mT/m) with integrated shims. A small transmit-receive ribbon solenoid RF coil (ID of 14 mm), which can be tuned either to ^1^H or ^19^F resonant frequency (i.e., 400.130 vs. 376.498 MHz) was used for all experiments. Coil geometry was adjusted individually to analyze sample size and shape to maximize filling factor and thus SNR values. Paravision 5.1 and Topspin 2.0 software was used to accomplish MR imaging and spectroscopy. Data was processed in Origin and ImageJ software.

The inversion recovery (IR) method was used to obtain the value of T_1_ relaxation time. Experimental points were measured for: 30,000, 20,000, 15,000, 10,000, 5000, 4500, 4000, 3750, 3500, 3250, 3000, 2000, 1000, 500, 100 ms inversion recovery times.

For the acquisition of axial ^1^H and ^19^F images, FLASH sequence was used (for ^1^H: echo time TE = 6 ms, flip angle FA = 30°, repetition time TR = 100 ms, number of acquisitions NEX = 4, total acquisition time TA = 51 s; for ^19^F: TE = 3.1 ms, FA = 15°, TR = 100 ms, NEX and TA given in the table below). The optimal FA (Ernst angle) was used, adjusted to the T_1_ and TR values ^19^F images were measured with several numbers of excitation to find the value that will give an acceptable compromise between SNR (signal-to-noise ratio) value and total acquisition time.

For the ^19^F MR measurements selected 5-FU nanocarriers were concentrated ~20 times to mimic an increased local concentration of ^19^F nuclei in the target region of the sample.

#### 3.3.5. Bioanalysis

##### Cells and Reagents Used in Biological Studies

The murine 4T1 mammary carcinoma (ATCC CRL-2539) was purchased from Dr. Gary Sahagian’s lab (Tufts University, Boston); murine undifferentiated colon carcinoma CT26 (ATCC CRL-2638) stably expressing human carcinoembryonic antigen (CT26-CEA), was a kind gift from Dr. Michał Bereta (Jagiellonian University, Kraków, Poland). The MTT reagent (3-[4,5-dimethylthiazol-2-yl]-2,5diphenyl tetrazolium bromide) and RNAase A were obtained from Sigma-Aldrich. Propidium iodide used for cell cycle analysis was purchased from BD Pharmingen.

##### Analysis of the Cytotoxic Activity of Encapsulated 5-Fluorouracil

The cells were grown in the incubator under standard conditions (5% CO_2_, 37 °C, >95% humidity) in the growth medium (DMEM containing 5% FBS; all reagents were from LONZA). Next, the cells were collected using trypsin (1% trypsin-0.53 mM EDTA solution; LONZA) and transferred in the growth medium to the 96-well plates at the density of 5 × 10^3^ cells per well and incubated overnight. Before experiments, the medium was discarded and replaced with 0.1 mL of fresh containing 10% FCS, antibiotics (penicillin 100 U/mL and streptomycin 100 μg/mL) and (1) 0.015 M NaCl, (2) empty nanocarriers, (3) 5-FU loaded nanocarriers, (4) 5-FU (solution in DMSO). Next, the cells were incubated for 48 h. The concentration of free or encapsulated fluorouracil was 2.56 µg/mL. All nanocarriers were suspended in 0.015M NaCl. The viability of the cancer cells exposed to empty nanocarrier, free or encapsulated 5-fluorouracil, was analyzed by the MTT method using a standard protocol.

##### Flow Cytometry Analysis of the Cell Cycle

The cells (4 × 10^4^) were transferred in 1 mL of growth medium into a 12-well plate and cultured for 24h. Then, the medium was discarded, and the cells were cultured for 48 h in 1 mL of fresh medium supplemented with 10% FCS, antibiotic and: (1) 0.0015 M NaCl, (2) NC 6 PEG (3) 5-FU NC 6 PEG), and (4) free 5-FU. Next, the cells were trypsinized, and an equal number of cells for each group was resuspended in 0.4 mL of PBS and fixed with 5 mL of cold 70% ethanol (in DI water). Finally, DNA was stained with PI using the standard protocol with modifications [49,57]. The PI-stained cells were analyzed using BD FACSCalibur flow cytometer and CellQuestPro software, Becton- Dickinson.

## 4. Conclusions

A novel method of preparation of multifunctional 5-Fluorouracil (5-FU) nanocarriers was developed. The procedure is based on the encapsulation of 5-FU loaded nanocores within polyaminoacids multilayer shells via a layer-by-layer approach. Biocompatibility of empty carriers, as well as activity of encapsulated 5-FU, were proven by in vitro tests. Moreover, the fluorine atom of 5-FU allows real-time observation of the developed system by fluorine magnetic resonance imaging (^19^F-MRI). Properties of our nanocarriers fulfill the requirements of the theranostic drug delivery system based on passive targeting via enhanced permeability and retention effect.

## Figures and Tables

**Table 1:** Characterization of selected 5-FU nanocarriers.

Name	Abbreviation	Size d	PDI	Zeta Potential
5-FU loaded nanocore	5-FU NC 1	80 nm	0.161	+72 mV
5-FU loaded 2 layered, PGA ended nanocarrier	5-FU NC 2 PGA	100 nm	0.149	−32 mV
5-FU loaded 6 layered PGA ended, nanocarrier	5-FU NC 6 PGA	145 nm	0.176	−36 mV
5-FU loaded 6 layered PEG ended nanocarrier	5-FU NC 6 PEG	205 nm	0.261	−5 mV

## Data Availability

Not applicable.
